# The Potential of Non-Provitamin A Carotenoids for the Prevention and Treatment of Non-Alcoholic Fatty Liver Disease

**DOI:** 10.3390/biology5040042

**Published:** 2016-11-08

**Authors:** Ana Gabriela Murillo, Diana M. DiMarco, Maria Luz Fernandez

**Affiliations:** Department of Nutritional Sciences, University of Connecticut, Storrs, CT 06269, USA; ana.murillo_solis@uconn.edu (A.G.M.); diana.dimarco@uconn.edu (D.M.D.)

**Keywords:** non-alcoholic fatty liver disease (NAFLD), carotenoids, lycopene, astaxanthin, lutein, zeaxanthin, hepatic steatosis

## Abstract

Non-alcoholic fatty liver disease (NAFLD) is an obesity-associated spectrum of comorbidities defined by the presence of metabolic dysfunction, oxidative stress, inflammation, and fibrosis in the liver. If left untreated, NAFLD can progress to cirrhosis, liver failure, or hepatocellular carcinoma. NAFLD is recognized as the most common liver disease in the United States, affecting around 30% of the population. Identification of dietary components capable of reducing or preventing NAFLD is therefore essential to battle this condition. Dietary carotenoids including astaxanthin, lycopene, lutein, and zeaxanthin have been demonstrated to be potent antioxidants as well as to exhibit anti-inflammatory effects. Many studies report the protective effect(s) of these carotenoids against different conditions such as atherosclerosis, diabetic complications, age-related macular degeneration, and liver diseases. In this review, we will focus on the effects of these carotenoids in the prevention or reduction of NAFLD as seen in epidemiological observations and clinical trials, as well as the suggested mechanism of action derived from animal and cell studies.

## 1. Introduction

Non-alcoholic fatty liver disease (NAFLD) is the broad spectrum of hepatic diseases ranging from non-alcoholic fatty liver (NAFL) or hepatic steatosis, to non-alcoholic steatohepatitis (NASH), which can further progress to more severe complications including fibrosis, cirrhosis, liver failure, hepatocellular carcinoma, and, ultimately, death [1,2,3,4].

NAFLD is the most common cause of abnormal liver function among adults in the United States [5,6]. The prevalence of this disease ranges between 25%–45% and it increases in parallel with other chronic comorbidities such as obesity, dyslipidemia, and Type 2 Diabetes Mellitus (T2DM). In fact, NAFLD is considered the hepatic manifestation of metabolic syndrome [7,8]. The incidence of NASH is less common, affecting an estimated 2%–3% of the general population and up to 37% of the morbidly obese [9,10]. However, to establish the prevalence of NASH in the general population is a challenge because the diagnostic requires histologic evaluation [6].

Although NAFLD is usually considered to exclusively affect adults, the rising prevalence of obesity and T2DM in children and adolescents over the last two decades has caused NAFLD to emerge as the leading cause of chronic liver disease in this segment of the population as well [11,12].

**Figure 1:**
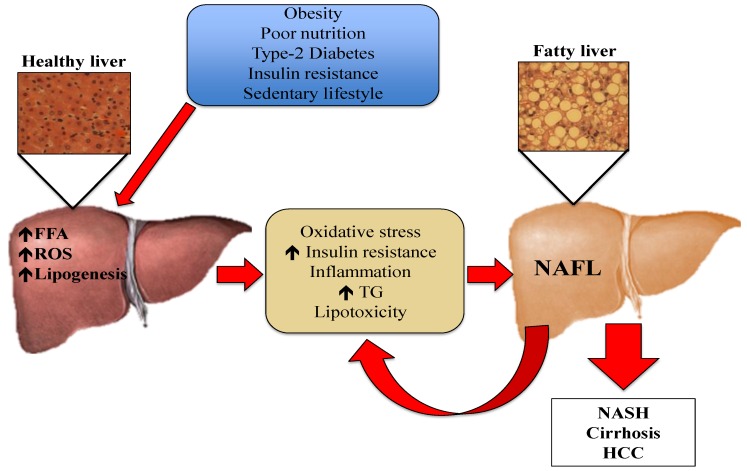
Pathogenesis of NAFL: Risk factors such as obesity, poor nutrition, Type-2 Diabetes, insulin resistance, and sedentary lifestyle cause an increment in the efflux of FFA from the adipose tissue to the liver. The increased FFA in hepatic tissue stimulates TG synthesis and accumulation. This excess of lipids induces an immune response, which causes the production of cytokines, ROS, and increased insulin resistance. Oxidative stress causes lipid peroxidation and tissue damage, amplifying the inflammatory response and leading to the progression of NAFLD. FFA: free fatty acids, HCC: hepatocellular carcinoma, NAFL: non-alcoholic fatty liver, NASH: non-alcoholic steatohepatitis, ROS: reactive oxygen species, TG: triglycerides.

One of the mechanisms underlying the development and progression of NAFLD is oxidative stress [7,13,14]. Oxidative stress is the imbalance between oxidants and antioxidants, potentially leading to cell and tissue damage. Although oxidants are a normal product of aerobic metabolism, they can be produced at elevated rates under certain pathophysiological conditions [15]. In the case of hepatic steatosis, the excess of lipids in the liver induces an innate immune response, with recruitment of immune cells such as macrophages and T-cells. This leads to the development of insulin resistance and the generation of reactive oxygen species (ROS) [16]. Although normal hepatic metabolism produces ROS, external factors also generate oxidative stress [14,17]. These ROS can induce lipid peroxidation and the disruption of proteins and DNA. Moreover, ROS induce necrosis and apoptosis of hepatocytes and amplify the inflammatory response, which can lead to fibrogenesis, a fundamental characteristic of the progression from NAFL to NASH [18]. Liver resident macrophages, also known as Kupffer cells, hepatic stellate cells, and endothelial cells are particularly sensitive to oxidative stress-related molecules [19]. Pro-inflammatory cytokines such as tumor necrosis factor α (TNF-α) can be produced in Kupffer cells in response to oxidative stress, and generate an inflammatory response or even promote apoptosis [14,18]. The mechanisms depicting the pathogenesis of NAFL are presented in Figure 1.

Standard dietary interventions for NAFLD include modifications of macronutrient intake such as carbohydrate and fat restriction; however, given the vital role of oxidative stress in the pathogenesis and development of liver diseases, various antioxidants have been proposed as preventative and therapeutic strategies. Emerging dietary therapies for NAFLD include the use of dietary components with antioxidant activity, such as Vitamins C and E, polyphenols, betaine, anthocyanins, curcumin, carotenoids, and resveratrol, which could counteract the damaging effects of ROS [9,14,20]. For example, in the National Health and Nutrition Examination Survey (NHANES) III, higher levels of serum carotenoids were associated with lower presence of apparent liver injury, as indicated by plasma concentrations of liver enzymes alanine aminotransferase (ALT) and aspartate aminotransferase (AST), suggesting a possible protective role of these compounds on NAFLD [21].

Carotenoids are a class of lipid soluble phytochemicals which are synthesized only by plants and some photosynthetic bacteria [22,23]. As part of the plant’s antioxidant machinery, carotenoids protect cellular structures against oxidative damage by scavenging singlet molecular oxygen and peroxyl radicals resulting from light exposure and normal plant metabolism [23,24,25,26]. As pigments, carotenoids are responsible for many of the red, orange, pink, and yellow colors of plant leaves, fruits, vegetables, and some birds, insects, fish, and crustaceans [25,27]. There are more than 700 naturally-occurring carotenoids identified, but only around 50 are present in detectable amounts within the human diet and just six represent more than 95% of plasma carotenoids [28,29]. Because carotenoids are lipophilic compounds, they can be stored in the body. The liver is the main storage site, which may explain why carotenoids have been studied in relation to hepatic health and disease [30].

Carotenoids can be classified as provitamin A or non-provitamin A. Provitamin A carotenoids can be metabolized into retinal and retinol, and therefore contribute to Vitamin A intake [31]. Examples of these include α-carotene, β-carotene, and α-cryptoxanthin [32]. Conversely, non-provitamin A carotenoids are those that do not exhibit Vitamin A activity. However, these have been associated with a variety of health benefits unrelated to Vitamin A function [29,33]. This group of carotenoids includes lycopene, lutein, zeaxanthin, and astaxanthin [34,35]. In this review, we focused on the potential of these non-provitamin A carotenoids in the prevention or treatment of NAFLD.

## 2. Astaxanthin

Astaxanthin is an oxygenated carotenoid found in aquatic animals living in astaxanthin-rich environments [36]. This pigment is responsible for the characteristic pink and red color of seafood such as salmon, trout, shrimp, lobster, and fish eggs [37]. Due to its long chain with conjugated double bonds and keto (C=O) moieties on each ionone ring, astaxanthin is a potent antioxidant that scavenges mostly peroxyl radicals, protecting fatty acids and biological membranes against lipid peroxidation [37,38,39]. For this reason, the potential of this carotenoid in ameliorating oxidative stress-induced diseases such as NAFLD has been studied [36].

Ni et al. [40] evaluated the effects of astaxanthin on lipid accumulation in two different mouse models. C57BL/6J mice and ob/ob mice were fed normal chow (10% kcal from fat) or a high-fat (60% of kcal from fat) diet with or without 0.02% astaxanthin for 10 weeks. Astaxanthin administration reduced hepatic steatosis and triglyceride (TG) accumulation significantly in both types of mice, with no alteration in body weight or adiposity. In the same study, the effects of astaxanthin were further compared in vitro with Vitamin E, which is one of the standard antioxidant treatments for NAFLD. Primary hepatocytes were incubated with either astaxanthin or α-tocopherol in the presence of oleic acid to promote lipid accumulation. The results showed that incubation with astaxanthin, but not α-tocopherol, resulted in a dose-dependent decrease in TG accumulation in primary hepatocytes. The mechanism by which astaxanthin decreased lipid accumulation in hepatocytes is suggested to be a reduction in fatty acid uptake as shown by a dose-dependent decrease in expression of cluster of differentiation 36 (CD36) with astaxanthin [38].

Another experiment was conducted to evaluate the appropriate dose of astaxanthin to prevent NASH in mice [38]. C57BL/6J mice were fed six different diets for 12 weeks: normal chow, chow containing 0.0067% or 0.02% astaxanthin, high-fat, high-cholesterol, and cholate diet, or high-fat, high-cholesterol diet with 0.0067% or 0.02% astaxanthin. Treatment with astaxanthin showed decreased concentrations of plasma liver enzymes in a dose-dependent manner, suggesting that 0.02% astaxanthin was the most effective dose. This concentration of astaxanthin was further used to compare its effects with Vitamin E. Astaxanthin was found to decrease plasma TG, total cholesterol (TC), and non-esterified fatty acid (NEFA) concentrations as well as AST and ALT activity when compared to control mice. Vitamin E only showed an improvement in plasma lipids but not in liver enzymes or NEFA concentrations [38]. Histological analysis revealed that astaxanthin intake resulted in lower lipid accumulation compared to control mice, with Vitamin E having less of an effect. In addition, astaxanthin supplementation suppressed the expression of lipogenic genes whereas Vitamin E did not. Lastly, astaxanthin and Vitamin E resulted in lower inflammation and fibrosis in hepatic tissue. However, the effect of astaxanthin was more prominent when compared to the effect of Vitamin E. In summary, the results presented by these investigators not only show that astaxanthin supplementation prevents NAFLD and NASH in mice, but also that this carotenoid is more effective than the standard antioxidant treatment for NAFLD.

In another study with astaxanthin and NAFLD, conducted by Yang et al. [38], male C57BL/6J mice aged six weeks were randomly assigned to one of four groups (*n* = 8 per group): high-fat diet control (35% kcal from fat, w/w) or high-fat diet supplemented with 0.003%, 0.01%, or 0.03% (w/w) of astaxanthin. These doses are equivalent to 20, 66, and 200 mg/day in humans, respectively. The results showed that the highest dose, 0.03%, was effective in significantly reducing plasma TG concentrations when compared to the control group, suggesting that this is the appropriate dose for reversion of diet-induced hypertriglyceridemia. Further analyses were therefore performed only with this group. Although there was no significant difference in the expression of lipogenic genes between the supplemented group and controls, there was an increase in the mRNA levels of acyl-CoA oxidase (ACOX) 1, an enzyme involved in fatty acid oxidation that has been shown to be altered in NAFLD [41]. This result suggests that astaxanthin induces fatty acid catabolism. ACOX1 is a target gene of peroxisome proliferator-activated receptor (PPAR) α, which regulates the expression of key proteins involved in mitochondrial and peroxisomal β-oxidation; it has been shown that PPARα can be activated by astaxanthin [42].

Because astaxanthin, as other carotenoids, is known to have an antioxidant effect, this study also examined whether supplementation with astaxanthin could impact nuclear factor (erythroid-derived 2)-like 2 (Nrf-2). Nrf-2 is referred to as the “master regulator” of the antioxidant response, modulating the expression of genes related to antioxidant enzymes, immune, and inflammatory responses [43]. The results showed a significant increase in the mRNA expression of Nrf-2 as well as its downstream genes, including superoxide dismutase (SOD) 1, glutamate–cysteine ligase regulatory subunit, and glutathione peroxidase (GPx) 1 [36].

In addition to its antioxidant capacity, one mechanism by which astaxanthin may protect the liver is via modulating macrophage polarization. Macrophages are components of the immune system that have a role in many aspects of an organism’s biology including development, homeostasis, repair, and the immune response [44]. These cells respond to diverse environmental signals by expressing a range of functional phenotypes. Two distinct macrophage activation states have been recognized, M1 and M2 [45]. M1 macrophages are potent effector cells that kill microorganisms and produce proinflammatory factors, including nitric oxide (NO), TNF-α, interleukin (IL)-6, and IL-12. In contrast, M2 macrophages produce anti-inflammatory mediators such as IL-10, transforming growth factor β (TGF-β), and IL-1 receptor antagonist (IL-1Ra) [46].

In the liver, hepatic macrophages, which consist of resident Kupffer cells and recruited bone marrow-derived macrophages, are the major cells that produce inflammatory mediators, such as TNF-α and IL-1β, which are directly related to insulin resistance and the progression from NAFL to NASH [47,48]. Some authors have suggested that Kupffer cells are a target of astaxanthin; supplementation with this carotenoid may inhibit M1 activation or drive M2 activation [47]. Astaxanthin supplementation also has decreased the proportion of M1 Kupffer cells in diet-induced NASH in both genetically (ob/ob) and diet-induced obese mice [40].

## 3. Lutein and Zeaxanthin

Lutein and its isomer zeaxanthin are non-provitamin A carotenoids that belong to the xanthophyll or oxycarotenoid family. These have the peculiarity of having two hydroxyl groups in their structure, which determines their specific behaviors in the body [22]. In food, lutein is particularly abundant in green leafy vegetables whereas zeaxanthin is mostly found in yellow foods such as corn and corn products [49]. Another good source of these carotenoids is egg yolk; although the concentration in egg yolk is relatively low compared to vegetables, recent data suggest that lutein and zeaxanthin from this food source are highly bioavailable [50,51,52,53].

The effect of lutein and zeaxanthin on eye health has been widely described, as these compounds are selectively taken up and accumulated in the macular region of the retina in primates. Here, these carotenoids are referred to as the macular pigment and they provide protection against oxidative damage [52,54].

The antioxidant properties of these two carotenoids are based in two main mechanisms. Because lutein and zeaxanthin have nine conjugated double bonds in the polyene chain, these can absorb light at 450 and 451 nm, respectively [55]. This specific wavelength corresponds to blue light, which is photo-oxidative and damaging to the eye tissue. The second mechanism is by directly quenching free radicals, especially singlet oxygen (^1^ O_2_) [54]. For this reason, numerous studies have linked the intake of lutein and zeaxanthin with the prevention of age-related macular degeneration [56]. In addition to being important to maintain eye health, other potential benefits of lutein and zeaxanthin have been proposed. For example, these compounds may enhance immune function and protect against cancer, cardiovascular disease, and oxidative stress related-diseases, including NAFLD [33,50,57]. Epidemiological studies have shown that plasma lutein and zeaxanthin are inversely associated with the prevalence of NAFLD in middle-aged and elderly Chinese populations [21].

The effects of lutein on cholesterol-induced liver damage was tested by Kim et al. using a guinea pig model [58]. Guinea pigs were fed a hypercholesterolemic (0.25% (wt/wt) cholesterol) diet, which has been shown to induce hepatic steatosis [59], with or without 0.1% (wt/wt) lutein, equivalent to 3 mg lutein/day, for 12 weeks. Livers from animals supplemented with lutein had 43% lower hepatic free cholesterol when compared to controls. Similarly, concentrations of hepatic malondialdehyde (MDA), a commonly used indicator of lipid peroxidation [60], were lower in the lutein group than in the control group. Lutein-fed guinea pigs also had lower protein concentrations of TNF-α and lower nuclear factor κB (NF-κB) DNA binding activity than the control group, suggesting that lutein also has anti-inflammatory effects in hepatic tissue [58].

Similar results were obtained with a nanoemulsion of lutein (3.5 mg/day) added to the diet using the same cholesterol challenge for six weeks in guinea pigs [57]. The nanoemulsion was used to improve the bioavailability of lutein, which was shown by increased concentrations of this carotenoid in both plasma and liver when compared to the same dose of powdered lutein. The guinea pigs fed with the nanoemulsion had 24% lower hepatic steatosis scores as assessed histologically, 31% lower hepatic TC accumulation, and 64% lower plasma ALT activity than did the control group, and overall had better results when compared to powdered lutein. Hepatic oxidized low-density lipoprotein (LDL) was 55% lower in both supplemented groups when compared to controls. These results suggest that the protective effects of lutein in the liver could be dose-dependent [61].

The effects of lutein on NAFLD have also been evaluated in Sprague-Dawley rats. In a study by Qiu et al. [62], forty animals were randomly divided into two groups. One group was fed a normal diet (*n* = 8), while the rest of the rats were fed a high-fat diet (*n* = 32) to induce disturbances in lipid metabolism. On the tenth day, plasma lipids were measured and the rats fed the high-fat diet were then divided into four groups based on plasma cholesterol concentrations and given 0, 12.5, 25, or 50 mg/kg body weight/day of lutein via oral gavage for the next 45 days. Hepatic TC was lowest with the highest dose of lutein and hepatic TG was also lower with 12.5 and 25 mg of lutein. Similarly, lutein supplementation effectively reduced the increase in serum ALT activity caused by the high-fat diet, suggesting a protective effect against fat-induced damage. The increases in blood glucose and insulin due to the high-fat diet were ameliorated by all doses of lutein for glucose, and with 25 and 50 mg/kg lutein for insulin, while the homeostatic model assessment index was effectively attenuated by lutein supplementation at all doses. In addition, 45 days of supplementation with lutein resulted in upregulation of insulin receptor substrate 2, phosphoinositide 3-kinase, and glucose transporter 2 when compared to the control, denoting better insulin sensitivity [58]. Further, because PPARα plays an important role in lipid metabolism, this study also investigated the effects of lutein on PPARα expression and found that the high-fat diet significantly inhibited the expression of PPARα, which was restored with lutein supplementation [62].

Zeaxanthin has also been tested individually in NAFLD models. In a study by Chamberlain et al. [63], 24 weanling male Mongolian gerbils were divided into four groups (*n* = 6 per group). One group was fed a standard control diet and the other three groups were given methionine-choline-deficient diets to induce NASH. Two of the methionine-deficient groups were also given gelatin containing 12.5 mg/kg or 25 mg/kg of zeaxanthin. After six weeks, no differences were observed in gross histopathology between supplemented groups and the controls. However, the choline deficient group supplemented with 25 mg/kg of zeaxanthin had no fibrosis. Furthermore, both zeaxanthin treatments resulted in lower lipid hydroperoxides compared to the control in a dose-dependent manner, suggesting that the mechanism of action of zeaxanthin is related to its antioxidant capacity.

## 4. Lycopene

Lycopene is the acyclic isomer of β-carotene. This highly unsaturated, non-oxygenated carotenoid is responsible for the red color in fruits and vegetables. Tomatoes and tomato-based products are the most common sources of lycopene in the human diet and account for more than 85% of the dietary intake of this carotenoid in North America [23,64,65].

Epidemiological studies have shown an inverse relationship between tomato intake and a number of chronic diseases and certain types of cancer, and researchers have suggested that this reduced risk is due to higher concentrations of lycopene in plasma and the beneficial effects that this may provide [23,66,67]. Lycopene is one of the most potent dietary antioxidants. Its long, acyclic polyene chain gives it a singlet oxygen quenching capacity higher than that of β-carotene and α-tocopherol [65,66,68]. Beyond antioxidant capacity, there are many other non-antioxidant potential mechanisms by which lycopene can protect against chronic diseases, including regulation of gene expression, gap-junction communication, anti-proliferative capacity, and hormone and immune modulation, among others [23,66,69]. For this reason, lycopene is one of the most studied carotenoids in the prevention and treatment of NAFLD [1].

Bahcecioglu et al. [70] investigated the preventative effect of lycopene on high-fat diet-induced NASH in Sprague-Dawley rats. Forty animals were divided into four groups and fed either a standard diet with 35% kcal from fat (control), a high-fat diet with 71% kcal from fat, and two groups were fed a high-fat diet plus lycopene at doses of 2 mg/kg or 4mg/kg body weight for a period of six weeks. Lycopene was mixed with olive oil and administered three days/week by gastric gavage. Rats fed the high-fat diet showed higher serum activity of the liver enzymes ALT, AST, and alkaline phosphatase (ALP), which is indicative of liver injury [71], as well as plasma TG and TC, insulin resistance, and higher serum and liver MDA and serum TNF-α when compared to the control group, indicating oxidative stress and inflammation caused by the excess dietary fat. The groups supplemented with lycopene had a restoration of liver enzyme activity to values similar to controls in a dose-independent manner. Similarly, serum and hepatic ALT, plasma TG, and serum TNF-α were significantly lower in both groups treated with lycopene when compared to control rats. Histological evaluation showed lower steatosis and inflammation in rats supplemented with lycopene compared to high-fat diet fed rats (*p* < 0.05), however, no differences were found in ballooning degeneration, fibrosis, or necrosis [70].

These data indicate that supplementation with 2 or 4 mg/kg body weight of lycopene can reduce high-fat diet-induced oxidative stress and liver damage, suggesting a protective role of this carotenoid in the development of NAFLD.

A similar study conducted by Wang et al. [72] tested the effects of purified lycopene and lycopene in tomato extract on the development of NASH-promoted hepatocarcinogenesis. Sprague-Dawley rats were given a single intraperitoneal injection of 30 mg diethylnitrosamine (DEN)/kg body weight. DEN is a representative chemical carcinogen commonly used to induce tumors in animal models [73]. After recovery from DEN injection, animals were randomly assigned to one of six groups (*n* = 8 per group) and fed ad libitum either a control diet (35% kcal from fat) or high-fat diet (71% kcal from fat) with or without pure lycopene (water-soluble beadlets, 95% all-trans isomers) or tomato extract supplements for six weeks.

Lycopene concentration was measured in both plasma and liver via high performance liquid chromatography. Both supplemented groups showed higher concentrations of hepatic lycopene when compared to supplemented controls, while no significant difference was found within supplemental groups. The effect of fat was not observed in plasma lycopene concentrations. These results suggest that both pure lycopene and tomato extract have the same effect in increasing plasma and tissue concentrations of this carotenoid and that the addition of extra fat in the meal plays a major role in the absorption of lycopene. This phenomenon has been described before, as fat is required for satisfactory absorption of carotenoids [74]. These differences in lycopene concentrations as a result of dietary fat were observed in liver and not in plasma, likely because at steady state, plasma carotenoids represent only 1% of the total body content of carotenoids, whereas the highest concentration can be found in the liver [30].

Oxidative stress was measured by quantifying lipid peroxidation end products such as MDA and 4-hydroxynonenal (4-HNE). Dietary supplementation with either lycopene or tomato extract significantly decreased high-fat diet-induced generation of both products, demonstrating the antioxidant capacity of lycopene. It is important to note, however, that in the case of tomato extract the antioxidant activity cannot be attributed to lycopene alone. Tomatoes have other bioactive components that could contribute to this effect, such as Vitamin C, Vitamin E, flavonoids, and other carotenoids [75]. Similarly, Wang’s study showed that tomato extract, but not purified lycopene, reduced high-fat diet-induced liver inflammation by decreasing both the number of inflammatory foci observed in histological evaluation and mRNA expression of multiple pro-inflammatory cytokines such as IL-6, IL-1β, and IL-12. These effects are possibly due to a synergistic effect between the bioactive components in tomatoes. However, purified lycopene resulted in higher concentrations of nuclear protein Nrf-2 when compared to controls. This effect was not observable with the tomato extract groups, suggesting that lycopene has a specific mechanism for protecting against oxidative stress [72].

Other studies have evaluated the effects of lycopene not in preventing, but in treating NAFLD. A recent study used lycopene supplements in Sprague-Dawley rats with high-fat diet-induced NAFLD [76]. The purpose of this study was to assess if lycopene supplementation would have synergistic effects with a control diet in reversing hepatic steatosis and hepatic morphological changes produced by a high-fat diet after NAFLD has developed. The animals were divided in two experimental groups, control (*n* = 22) and high-fat diet (*n* = 34). After four weeks of a high-fat diet some animals were sacrificed for analysis. There was an increase in liver weight and hepatic lipids, and higher concentrations of TC and TG. Impaired glucose tolerance, which is common in the development of NAFLD due to insulin resistance [4], was also observed after a sucrose charge in the high-fat diet group. Similarly, high-fat diet-fed animals had higher serum ALT and AST activities, lower activities of antioxidant enzymes SOD and GPx, and higher concentrations of MDA. Morphological changes observed in the livers of the rats fed a high-fat diet included hepatocyte swelling and macrovesicular steatosis with numerous cytoplasmic vacuoles. The average steatosis score for these animals was significantly higher than that for the rats fed the control diet [76].

When the remaining rats from the high-fat diet group were returned to a normal diet with or without lycopene (20 mg/kg body weight/day of lycopene given via oral gavage), TC and high-density lipoprotein (HDL)-cholesterol concentrations returned to values comparable to controls. However, LDL-cholesterol concentrations returned to normality only with diet and lycopene supplementation, not with diet alone. Similarly, liver weight and hepatic TC were only restored completely when lycopene was added to the diet. Furthermore, the addition of lycopene to the control diet helped to revert the morphological changes seen in the histological evaluation from 90% of affected cells to less than 50%. SOD and GPx activities were comparable to those observed in the control group only with lycopene supplementation, which suggests that the mechanism by which lycopene exerts its protective effects is by reducing oxidative stress. The results observed in this particular study highlight the fact that diet control is crucial to the treatment of NAFLD. However, bioactive antioxidant components such as lycopene could be used in addition to dietary interventions to accelerate the recovery from and reversion of high-fat diet-induced liver damage.

It is important to understand that despite the fact that antioxidants are protective against NAFLD progression, this disease is more complex than a redox imbalanced state because it disturbs several metabolic systems in the liver that alter lipid and glucose metabolism [77,78]. To assess other possible mechanisms beyond antioxidant activity of lycopene, Bernal et al. [77] evaluated the effects of lycopene from tomato juice on liver metabolism of hypercholesterolemic and high-fat diet-fed Sprague–Dawley rats. Animals were assigned randomly to one of four groups (*n* = 5 per group): normal diet and water, normal diet and tomato juice, high-fat diet and water, or high-fat diet and tomato juice for five weeks. Based on juice intake, the animals consumed between 3.1–3.5 mg/day of lycopene. However, since the presence of fat allows for higher absorption of carotenoids, the high-fat diet group exhibited five times higher concentrations of lycopene in the liver.

Both high-fat diet groups exhibited characteristics of hepatic steatosis, such as swelling of hepatocytes and fat accumulation. However, urinary isoprostanes, a marker of oxidative stress, were significantly increased only in the diet with no lycopene. In addition, a significant improvement in plasma HDL-cholesterol and TG concentration was observed in the high lutein group compared with control animals.

An important finding in this research is that the tomato juice had effects on liver metabolites that had no relation to the redox state. For example, hepatic amino acids increased, tending toward those seen in a normal diet and metabolic pattern, suggesting that this food is protective by more than just its antioxidant mechanism.

Another effect of the tomato juice independent of antioxidant activity was increased expression of genes related to fatty acid transport, lipid hydrolysis, and mitochondrial and peroxisomal beta oxidation [79]. Further, as reported by Martín-Pozuelo et al. [79], plasma pro-inflammatory vascular cell adhesion molecule 1 (VCAM-1), a marker of atherosclerosis, was lower in the high-fat diet with lutein group when compared to the control, although this could be associated with higher HDL-cholesterol and not directly with tomato juice. Studies have shown that HDL can reduce endothelial cell expression of VCAM-1 [80].

Although the authors of these two papers [75,76] attribute the protective effects of tomato juice to lycopene, it is pertinent to remark that the treatment given to the rats was not lycopene alone, and therefore the observed effects could be due to other bioactive components present in tomatoes or a synergistic effect between lycopene and these compounds. However, studies with lycopene alone have shown that this carotenoid does play a role in the regulation of hepatic metabolism. In a study conducted by Ahn et al. [81], male C57BL/6J mice were divided into three groups (*n* = 10 per group): mice given a normal-fat diet (11.69% kcal from fat), mice given a high-fat diet (49.29% kcal from fat), and mice fed the same high-fat diet with 0.05% lycopene for eight weeks.

As expected, mice fed the high-fat diet exhibited signs of NAFLD such as hepatomegaly and steatosis with ballooning degeneration and increased concentrations of hepatic TC and TG compared to control mice. However, lycopene supplementation effectively protected against high-fat diet-induced hepatic lipid accumulation. Similarly, lycopene reversed the effects of a high-fat diet on PPARα and PPARγ, two major transcription factors involved in the regulation of hepatic lipid metabolism, and increased the expression of apolipoprotein A-IV (ApoA-IV), a major component of HDL particles known to contribute to HDL antioxidant activity [82,83]. The proposed mechanism by which lycopene can exert these effects is via microRNA (miRNA) modulation. These are small non-coding RNAs that are involved in post-transcriptional regulation of their target genes, regulating lipid synthesis, fatty acid oxidation, cholesterol metabolism, and lipoprotein formation and secretion [84,85].

In this study, the high-fat diet caused a decrease of miRNA-21, which inhibits expression of fatty acid binding protein 7 (FABP7). FABP7 promotes hepatic lipid accumulation; Hepa 1–6 cells lacking FABP7 showed a decrease in lipid concentration. Treatment with lycopene prevented the decrease of miRNA-21 induced by high-fat diet, thereby suppressing FABP7 and, thus, hepatic steatosis [81].

A summary of studies examining the role of these non-provitamin A carotenoids on NAFLD are described in Table 1.

## 5. Conclusions

**Figure 2:**
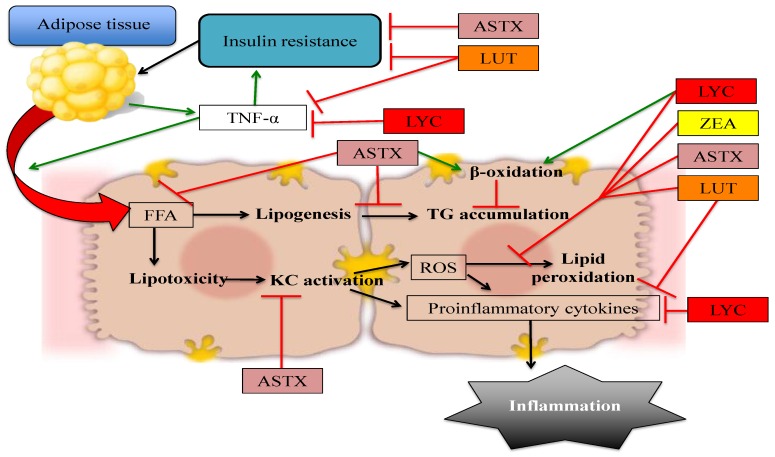
Non-provitamin A carotenoids have the potential to prevent or treat NAFLD by affecting different pathways: The quenching of ROS, thus decreasing oxidative stress and lipid peroxidation. However, other mechanisms include decreasing hepatic FFA influx, modulating expression of lipogenic and lipolytic genes, improving insulin signaling, and directly reducing inflammation. Red arrows denote blocked or decreased pathways whereas green arrows represent increased or promoted pathways. ASTX: astaxanthin, FFA: Free fatty acids, KC: Kupffer cells, LUT: lutein, LYC: lycopene, ROS: reactive oxygen species, TNF-α: tumor necrosis factor α, ZEA: zeaxanthin.

The available evidence regarding the potential use of non-provitamin A carotenoids in the prevention and treatment of NAFLD suggests that these compounds are effective in decreasing lipid accumulation, insulin resistance, oxidative stress, and inflammation in hepatic tissue, and therefore could be used as a dietary alternative to ameliorate the characteristic damage observed in NAFL and prevent its progression to more complicated stages such as NASH and liver fibrosis. The effects of specific mechanisms by which non-provitamin A carotenoids protect against NAFLD are depicted in Figure 2.

Although the classic mechanism by which non-provitamin A carotenoids can protect against NAFLD and other chronic diseases is by quenching ROS and decreasing oxidative damage, more complex pathways related to gene expression, inflammation, and macrophage plasticity are being elucidated. This suggests that each carotenoid can interfere differently with the development of NAFLD, creating a potential synergistic effect if they are consumed together. Therefore, a dietary approach that could be used in NAFLD patients is the Mediterranean diet, which is characterized by a high intake of fruits and vegetables that are natural sources of carotenoids, among other bioactive components [86]. Several studies have found that the adherence to the Mediterranean diet is related to a low incidence of chronic diseases, including NAFLD. It is hypothesized that the presence of large amount of antioxidants, such as carotenoids, in this diet is likely responsible for this correlation [86,87].

However, more epidemiological data is needed to assess whether there could be a specific recommended intake of dietary carotenoids to prevent NAFLD or if this protection can be only achieved through pharmacological doses. In addition, it is important to note that carotenoids have low and inconsistent bioavailability and therefore, in the human situation, it could be more relevant to find an adequate plasma concentration of carotenoids, rather than a suggested intake.

Nevertheless, carotenoids, as with other bioactive components, most likely do not have a substantial effect on their own. The prevention of NAFLD should be achieved by a healthy diet and lifestyle that includes food groups and nutrients in the recommended proportions. The prevention of obesity, metabolic syndrome, and T2DM seems to be the most effective way to avoid NAFLD; perhaps these strategies could be complemented with the inclusion of natural sources of carotenoids in the diet.

## Figures and Tables

**Table 1:** Summary of studies where carotenoids had a beneficial effect on Hepatic Steatosis in Animal and Cell Models.

Carotenoid	Stage of Liver Disease	Study Model	Dietary Treatment	Dose	Duration of the Intervention	Main Outcomes	Reference
Astaxanthin	NASH	Male ob/ob mice and C57BL/6J mice	High-fat, high-cholesterol diet (60% kcal from fat +1.25% cholesterol)	0.0067 or 0.02% astaxanthin (w/w)	10 weeks	↓ Hepatic steatosis ↓ Hepatic lipid uptake and accumulation ↓ Plasma ALT, AST, TG, TC, and NEFA ↓ Hepatic lipid peroxidation ↓ Hepatic lipogenic gene expression ↑ Hepatic insulin sensitivity ↓ Hepatic inflammation and fibrosis	[40]
Astaxanthin	NAFLD	Male C57BL/6J mice	High-fat diet (35% w/w)	0.003, 0.01, or 0.03% astaxanthin (w/w)	12 weeks	↓ Plasma ALT and AST (with 0.03%) ↑ Hepatic FA β-oxidation ↓ Hepatic steatosis ↑ Hepatic antioxidant enzyme expression	[38]
Lutein	Hepatic steatosis	Male Hartley guinea pigs	Hypercholesterol-emic diet (0.25% w/w)	0.1% supplemental lutein	12 weeks	↓ Hepatic free cholesterol ↓ Hepatic MDA and TNF-α ↓ Hepatic NF-ĸB p65 DNA Binding	[58]
Lutein	NAFLD	Male Sprague-Dawley rats	High-fat diet (33% kcal from fat)	12.5, 25, or 50 mg/kg BW/day	10 days HFD, then 45 days of HFD + lutein	↓ Hepatic TC and TG ↑ Serum HDL-cholesterol ↓ Serum ALT ↑ Hepatic insulin sensitivity ↑ Hepatic FA catabolism	[62]
Lycopene	NAFLD	Male C57BL/6J mice	High-fat diet (49.29% kcal from fat)	0.05% lycopene	8 weeks	↓ Hepatic lipids, TG, and TC ↑ Hepatic PPARα, CPT1α, LCAD, and ApoA4 expression ↓ Hepatic PPARγ and FASN expression ↓ Hepatic lipogenesis ↑ Hepatic fatty acid β-oxidation ↓ Hepatic FABP7 expression via ↑ miRNA-21	[81]
Lycopene	NASH	Male Sprague-Dawley rats	High-fat diet	2 or 4 mg/kg BW, given 3× per week	6 weeks	↓ Serum ALT (both doses), glucose (with 2 mg/kg) TG (with 4 mg/kg) ↓ Serum MDA ↓ Serum TNF-α ↑ Hepatic glutathione ↓ Hepatic steatosis and inflammation ↓ Hepatic stellate cell activation	[70]
Lycopene	NAFLD	Male Sprague-Dawley rats	Hypercholesterol-emic/high-fat diet	3.15–3.5 mg/day lycopene from tomato juice	5 weeks	↓ Plasma TG ↑ Plasma HDL cholesterol ↓ Plasma VCAM ↑ Hepatic amino acids ↓ Urinary isoprostanes ↑ NAD/NADH ratio and redox balance ↑ Hepatic expression of genes related to FA transport, hydrolysis, and β-oxidation	[77,79]
Lycopene	NAFLD	Male Sprague-Dawley rats	High-fat diet (71% kcal from fat)	20 mg/kg BW/day supplemental lycopene	4 weeks high-fat diet then 4 weeks normal chow diet + lycopene	↓ Liver weight ↓ Serum LDL-cholesterol ↓ Hepatic TC ↑ Antioxidant enzyme activity ↓ Hepatic steatosis	[76]
Lycopene	NASH-promoted hepato-carcinogenesis	Male Sprague-Dawley rats	High-fat diet (71% kcal from fat)	15 mg/kg BW/day all-*trans* lycopene supplement or lycopene from tomato extract	6 weeks	↓ Cell growth and replication ↓ Precancerous lesions ↓ Inflammatory cytokine expression ↓ Oxidative stress	[72]
Zeaxanthin	NASH	Male Mongolian gerbils	Methionine- and choline-deficient diet	0, 12.5 or 25 mg/kg zeaxanthin	6 weeks	↓ Liver fibrosis (at highest dose) ↓ Hepatic lipid hydroperoxides	[63]

Apo: apolipoprotein; ALT: alanine aminotransferase; ALP: alkaline phosphatase; AST: aspartate aminotransferase; BW: body weight; CPT1α: carnitine palmitoyltransferase 1α; FA: fatty acid; FABP7: fatty acid binding protein 7; FASN: fatty acid synthase; HDL: high density lipoprotein; LCAD: long-chain acyl-CoA dehydrogenase; LDL: low density lipoprotein; MDA: malondialdehyde; miRNA: microRNA; NAD: nicotinamide adenine dinucleotide; NAFLD: non-alcoholic fatty liver disease; NASH: non-alcoholic steatohepatitis; NEFA: non-esterified fatty acid; NF-κB: nuclear factor κB; PPAR: peroxisome proliferator activated receptor; TC: total cholesterol; TG: triglycerides; TNF-α: tumor necrosis factor α; VCAM: vascular cell adhesion molecule. ^1^ ↑ indicates increase and ↓ indicates decrease.
